# Genetic Evidence for the Association between the Early Growth Response 3 (*EGR3*) Gene and Schizophrenia

**DOI:** 10.1371/journal.pone.0030237

**Published:** 2012-01-20

**Authors:** Rui Zhang, Shemin Lu, Liesu Meng, Zixin Min, Juan Tian, Robert K. Valenzuela, Tingwei Guo, Lifang Tian, Wenxiang Zhao, Jie Ma

**Affiliations:** 1 Department of Genetics and Molecular Biology, and Department of Epidemiology and Health Statistics, Xi'an Jiaotong University School of Medicine, Xi'an, Shaanxi, China; 2 Arizona Health Science Center, University of Arizona, Tucson, Arizona, United States of America; 3 Albert Einstein College of Medicine, New York, New York, United States of America; Wayne State University, United States of America

## Abstract

Recently, two genome scan meta-analysis studies have found strong evidence for the association of loci on chromosome 8p with schizophrenia. The early growth response 3 *(EGR3)* gene located in chromosome 8p21.3 was also found to be involved in the etiology of schizophrenia. However, subsequent studies failed to replicate this finding. To investigate the genetic role of *EGR3* in Chinese patients, we genotyped four SNPs (average interval ∼2.3 kb) in the chromosome region of *EGR3* in 470 Chinese schizophrenia patients and 480 healthy control subjects. The SNP rs35201266 (located in intron 1 of *EGR3*) showed significant differences between cases and controls in both genotype frequency distribution (*P* = 0.016) and allele frequency distribution (*P* = 0.009). Analysis of the haplotype rs35201266-rs3750192 provided significant evidence for association with schizophrenia (*P* = 0.0012); a significant difference was found for the common haplotype *AG* (*P* = 0.0005). Furthermore, significant associations were also found in several other two-, and three-SNP tests of haplotype analyses. The meta-analysis revealed a statistically significant association between rs35201266 and schizophrenia (*P* = 0.0001). In summary, our study supports the association of *EGR3* with schizophrenia in our *Han* Chinese sample, and further functional exploration of the *EGR3* gene will contribute to the molecular basis for the complex network underlying schizophrenia pathogenesis.

## Introduction

Schizophrenia (OMIM 181500) is a severe mental disorder with a lifetime risk of about 1%, characterized by hallucinations, delusions and cognitive deficits. Family, twin and adoption studies have consistently demonstrated an important genetic component to schizophrenia (∼80%), in addition to developmental and environmental influences [1], [2].

Identification of susceptibility genes associated with schizophrenia has been difficult. This is presumably due to the etiological complexity and abnormalities in development of this psychiatric disease. In spite of these difficulties, chromosome region 8p has been shown to be the most well-established association with schizophrenia. Recently, two genome-scan meta-analysis studies for psychiatric diseases have found strong evidence for the association of 8p with schizophrenia [3], [4]. In addition, a genome-scan study with a large sample of pedigrees has added further evidence for the hypothesis that at least one schizophrenia susceptibility gene may be located on chromosome 8p [5]. More recently, a number of liability genes located in chromosome 8p were found to be involved in the etiology of schizophrenia. *EGR3* is one of these compelling susceptibility genes that have been associated with schizophrenia in various ethnic populations [6], [7], [8], [9], [10], [11].

Early growth response (*EGR*) genes (*EGR1*, *EGR2*, *EGR3*, and *EGR4*) are a family of immediate early gene transcription factors that are important for neuronal responses [12]. All the family members share a highly conserved DNA-binding domain composed of three zinc-finger motifs. They play an important role in the mediation of gene transcription in neuronal development and are involved in the regulation of synaptic plasticity, learning and memory process [13], [14]. EGR1 and EGR3 are the most abundant EGR proteins in the brain, and their expression is up-regulated by synaptic activity in the brain [15]. EGR3 has an essential role in learning and memory processing of both short- and long-term hippocampus-dependent memory; it also mediates adaptation to stress and novelty [15], [16]. Therefore, *EGR3* is a compelling candidate gene for schizophrenia from functional and positional perspective.

Expression of *EGR3* has been reported to be significantly lower in postmortem hippocampus' of schizophrenic patients compared to the control subjects [17]. Expression of *EGR3* was also reported to be down-regulated in the dorsolateral prefrontal cortex of patients with schizophrenia. Moreover, pedigree and case-control analyses in a study utilizing a Japanese sample found that *EGR3* was significantly associated with schizophrenia; this result was also replicated in a Korean population study [8], [11]. However, subsequent studies utilizing a Japanese sample and a Chinese sample failed to replicate this finding [18], [19].

As a result of the “winner's curse” phenomena, a seemingly high proportion of false positive reports caused by the overestimation of genetic effects were published. A stringent criterion for interpreting association studies is that an association based on one study should be viewed as tentative until it has been independently replicated in at least one other study [20]. Hence, we performed a study to further explore the relationship between *EGR3* and schizophrenia in a case-control sample of Chinese.

## Results

**Figure 1:**
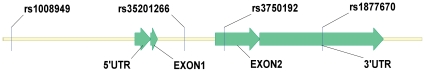
Organization and position of selected SNPs of *EGR3*.

We genotyped four SNPs (average interval ∼2.3 kb) in the chromosome region of *EGR3* in 470 Chinese schizophrenia patients and 480 control subjects (Figure 1). Hardy-Weinberg equilibrium (HWE) was tested separately in case and control samples. The genotype distribution of SNPs rs35201266, rs3750192, and rs1877670 were found to be in HWE (*P*>0.05) both in controls and cases. The genotype distribution of rs1008949 was not in HWE in patients (*P*<0.05). All SNPs were highly polymorphic in both groups.

### Single-marker analysis

The genotype and allele frequency distribution of all the SNPs in schizophrenia patients and controls are shown in Table 1. SNP rs35201266 showed significant differences between cases and controls both in the genotype frequency distribution (*X* ^2^ = 8.25, *P* = 0.016) and the allele frequency distribution (the A-allele: *X* ^2^ = 6.829, *P* = 0.009, odds ratio (OR) = 1.37, 95% confidence intervals (CI) = 1.07–1.75). Moreover, in the genetic model analysis (Table 2), a significantly positive result was observed for the A-allele of rs35201266 in the additive model (*X* ^2^ = 7.09, *P* = 0.0078, OR = 1.46), and weakly positive results were found in the dominant model (*X* ^2^ = 7.09, *P* = 0.0258, OR = 1.36, 95%CI = 1.04–1.79) and the recessive model (*X* ^2^ = 5.21, *P* = 0.0224, OR = 2.69, 95%CI = 1.11–6.50). After Bonferroni correction, the difference observed for rs35201266 (*P* = 0.036) in the allele frequency distribution and the additive model (*P* = 0.031) remained significant.

**Table 1:** Genotype and allele frequencies of the SNPs analyzed in cases and controls.

Marker	Genotype Distribution (%)					Allele Distribution (%)					
	Genotype	Case^a^	Control^a^	*X* ^2^	*P* value^b^	Allele	Case^a^	Control^a^	*X* ^2^	*P* value^b^	OR (95% CI)
rs1008949	CC	30.8(145)	32.3(155)	3.29	0.193	C	58.2(547)	57.1(548)	0.239	0.625	1.05 (0.87–1.26)
	CT	54.7(257)	49.6(238)			T	41.8(393)	42.9(412)			
	TT	14.5(68)	18.1(87)								
rs35201266	AA	3.8(18)	1.5(7)	8.25	**0.016**	A	20.1(189)	15.5(149)	6.829	**0.009**	1.37 (1.07–1.75)
	AG	32.6(153)	28.1(135)			G	79.9(751)	84.5(811)			
	GG	63.6(299)	74.4(338)								
rs3750192	GG	71.9(338)	66.3(318)	3.78	0.151	G	84.1(791)	81.3(780)	2.788	0.095	1.23 (0.96–1.57)
	GT	24.5(115)	30.0(144)			T	15.9(149)	18.7(180)			
	TT	3.6(17)	3.7(18)								
rs1877670	TT	30.0(141)	27.9(134)	0.55	0.758	T	55.3(520)	54.3(521)	0.211	0.646	1.04 (0.87–1.26)
	TC	50.6(238)	52.7(253)			C	44.7(420)	45.7(439)			
	CC	19.4(91)	19.4(93)								

a Number of alleles for each SNP is given in parentheses.

b Significant *P* value (<0.05) are in boldface.

**Table 2:** Analysis of the genetic models.

Marker	Genotype^a^			Dominant Model (Risk allele 1)			Recessive Model (Risk allele 1)			Additive Model (Risk allele 1)		
	11	12	22	*X* ^2^	*P* value^b^	OR (95% CI)	*X* ^2^	*P* value^b^	OR (95% CI)	*X* ^2^	*P* value^b^	OR^c^
**rs1008949**				0.23	0.6329	1.07 (0.81–1.41)	2.33	0.1272	0.76 (0.54–1.08)	0.26	0.6129	0.94
Cases	68	257	145									
Controls	87	238	155									
**rs35201266**				4.97	**0.0258**	1.36 (1.04–1.79)	5.21	**0.0224**	2.69 (1.11–6.50)	7.09	**0.0078**	1.46
Cases	18	153	299									
Controls	7	135	338									
**rs3750192**				3.57	0.0590	0.77 (0.58–1.01)	0.01	0.9134	0.96 (0.49–1.89)	2.26	0.1029	0.85
Cases	17	115	338									
Controls	18	144	318									
**rs1877670**				0.50	0.4790	0.90 (0.68–1.20)	0.00	0.9959	1.00 (0.72–1.38)	0.22	0.6389	0.96
Cases	91	238	141									
Controls	93	253	134									

a 1: Minor allele; 2: Major allele.

b Significant *P* value (<0.05) are in boldface.

c The software program Finetti (http://ihg2.helmholtz-muenchen.de/cgi-bin/hw/hwa1.pl) did not provide 95%CI.

### Haplotype analysis

**Figure 2:**
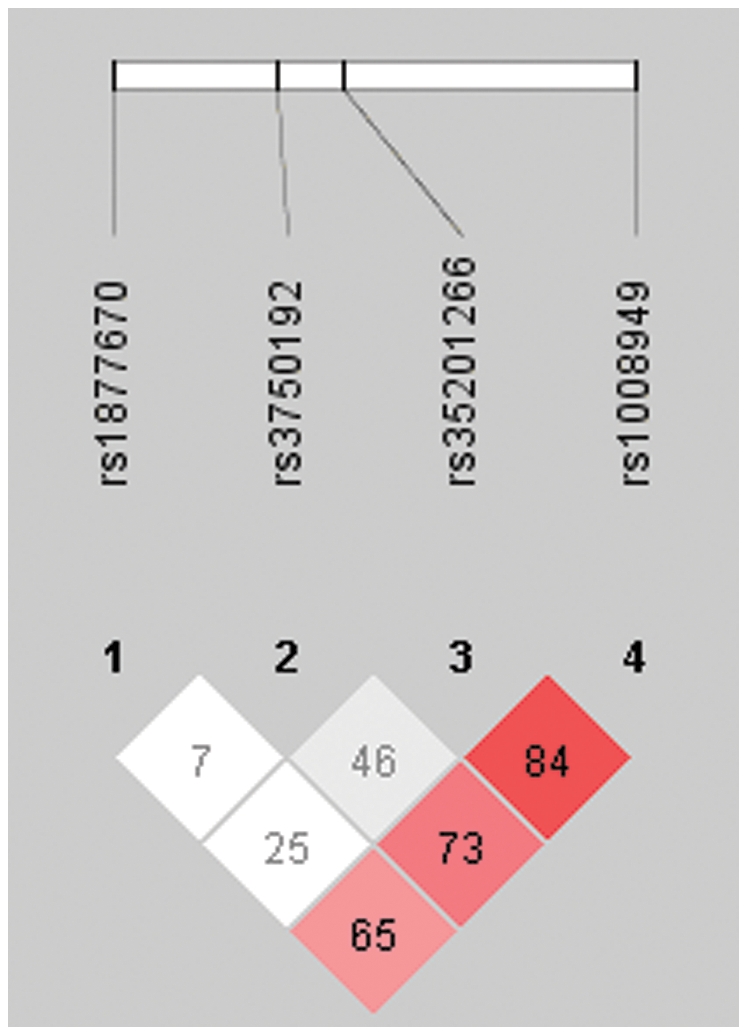
LD between markers genotyped in the *EGR3* gene locus in *Han* Chinese. LD structure (*D′*) between marker pairs is indicated by the shaded matrices. The figure was generated using HaploView 4.1.

Figure 2 presents the results of Linkage disequilibrium (LD) tests between pairs of SNP markers for the respective control groups. Owing to the medium LD (*D*′>0.8) between rs1008949 and rs35201266, we selected the rs35201266 as the relevant tagging SNP for haplotype analyses. Analysis of haplotype rs35201266-rs3750192 showed significant association with schizophrenia (global *P* = 0.0012). A significant difference was found for the common haplotype *AG* (*P* = 0.0005, OR = 1.77, 95% CI = 1.27–2.47) which was more prevalent in cases compared to controls (11.4% vs 6.8%). After correction for multiple testing (4 haplotypes and 4 allelic comparisons), the difference observed for the *AG* haplotype (*P* = 0.004) remained significant. In addition, several other two-, and three-SNP tests of haplotype association were also significant (Table 3). Haplotypes containing the A allele of rs35201266, were higher in frequency in cases compared to controls.

**Table 3:** Estimated frequency of haplotype and association significance.

No. of Markers	Haplotype	Global *P* a	Alleles Increasing in Cases	Estimated Haplotype Frequency(%)		*P* value^a^	OR (95% CI)
				Cases	Controls		
2	rs1008949-rs35201266	**0.0330**	C-A	17.8	14.5	**0.0445**	1.29 (1.00–1.65)
	rs35201266-rs3750192	**0.0012**	A-G	11.4	6.8	**0.0005**	1.77 (1.27–2.47)
3	rs1008949-rs35201266-rs3750192	**0.0246**	C-A-G	9.3	6.1	**0.0083**	1.59 (1.11–2.27)
	rs35201266-rs3750192-rs1877670	**0.0180**	A-G-C	7.1	4.5	**0.0135**	1.64 (1.08–2.47)

a Significant *P* values (<0.05) are in boldface.

### Age at onset (AAO) analysis

Since the marker rs35201266 showed significant association with schizophrenia, and schizophrenia onset is quite concentrated for people between the ages of 15 and 45 [21], we stratified the patients into 4 subgroups by AAO to examine if there were any clinical differences between the patients carrying allelic variants of rs35201266 (Table 4). However, no significant differences were found between the risk allele (A) carriers and the G homozygotes of rs35201266 (Global *P* = 0.2764). When the allele frequencies were compared in different subgroups between the A carriers and the G homozygous carriers, no significant differences were observed (*P*>0.1).

**Table 4:** Age at onset and gender analysis of rs35201266 (A/G) in cases.

Age at onset	Main effect		*P* value	OR (95% CI)	Gender	Main effect		*P* value	OR (95% CI)
	A carriers^a^	G homozygote^a^				A carriers^a^	G homozygote^a^		
<15	1.75 (3)	3.34 (10)	0.3119	0.52 (0.11–2.06)	Male	46.78 (80)	55.18 (165)	0.0794	0.71 (0.48–1.06)
15≦ and<30	78.36 (134)	78.34 (217)	0.1651	1.37 (0.86–2.19)	Female	53.22 (91)	44.82 (134)		
30≦ and<45	16.95 (29)	22.41 (67)	0.1586	0.71 (0.42–1.18)					
45≦	2.92 (5)	1.67 (5)	0.3656	1.77 (0.44–7.16)					
Global *P*			0.2764						

a Number of alleles for each SNP is given in parentheses.

### Gender analysis

Samples were stratified by gender according to the study of Zhang *et al*. [22] and marker rs35201266 was analyzed in patients. For the A carriers and the G homozygotes of rs35201266, there was no significant difference (*P* = 0.0794, OR = 0.71, 95% CI = 0.48–1.06) between males and females (Table 4).

### Comparing our results with previous association studies

A comparison of our results with the previous genetic studies have been performed, and the A-allele of rs35201266 showed the positive results in Chinese (*P* = 0.009), Korean (*P* = 0.0008), and a part of Japanese population (*P* = 0.0009) (Table 5). We also found that the MAF of rs35201266 was lower in the Chinese population compared to other populations (Table 5).

**Table 5:** Comparison of rs35201266 between current study and the previous studies.

Polymorphsim	Studies	Ethnicity	Sample	MAF^b^ in Control (Allele)	Risk Allele	*P* value^c^
rs35201266 (A/G)	Current Study	Chinese	Case-Control	0.155 (A)	A	**0.009**
	Kim et al., 2010 [11]	Korea	Case-Control	0.364 (A)	A	**0.0008**
	Yamada et al., 2007 [8]	Japanese	Family-trois	Unknown	A	**0.0009**
		Japanese	Case-Control	0.319 (A)	A	0.234
	Hapmap^a^	European	Control	0.392 (A)	Unknown	Unknown
		Nigeria (African)	Control	0.203 (A)	Unknown	Unknown

a Hapmap:http://hapmap.ncbi.nlm.nih.gov/.

b MAF: Minor Allele Frequency.

c Significant *P* value (<0.05) are in boldface.

When we compared our results with previous GWASs on schizophrenia (Table 6), the positive association between chromosome 8p and disease was identified at least in four different ethnic populations from three GWASs [23], [24], [25].

**Table 6:** Summary of GWA studies on chromosome 8p.

GWA Studies	Ethnicity	Sample	Region	Number of Positive Markers	Position (dbSNP 132)	*P* value
Shi et al., 2009 [27]	European Ancestry	Case-Control	8p23.3-8p21.2	22	549,908-25,655,470	<9.27×10^−4^
	African American	Case-Control	8p23.2-8p21.1	22	2,724,897–29,297,518	<9.26×10^−4^
Yamada et al, 2011 [28]	Japanese	Family-trios	8p23.3-8p21	37	1,817,045–27,577,392	<0.05
Ma et al., 2011 [29]	Chinese	Case-Control	8p23.1-8p22	7	10,022,938–10,062,543	<5.0×10^−5^

### Meta-analysis

**Figure 3:**
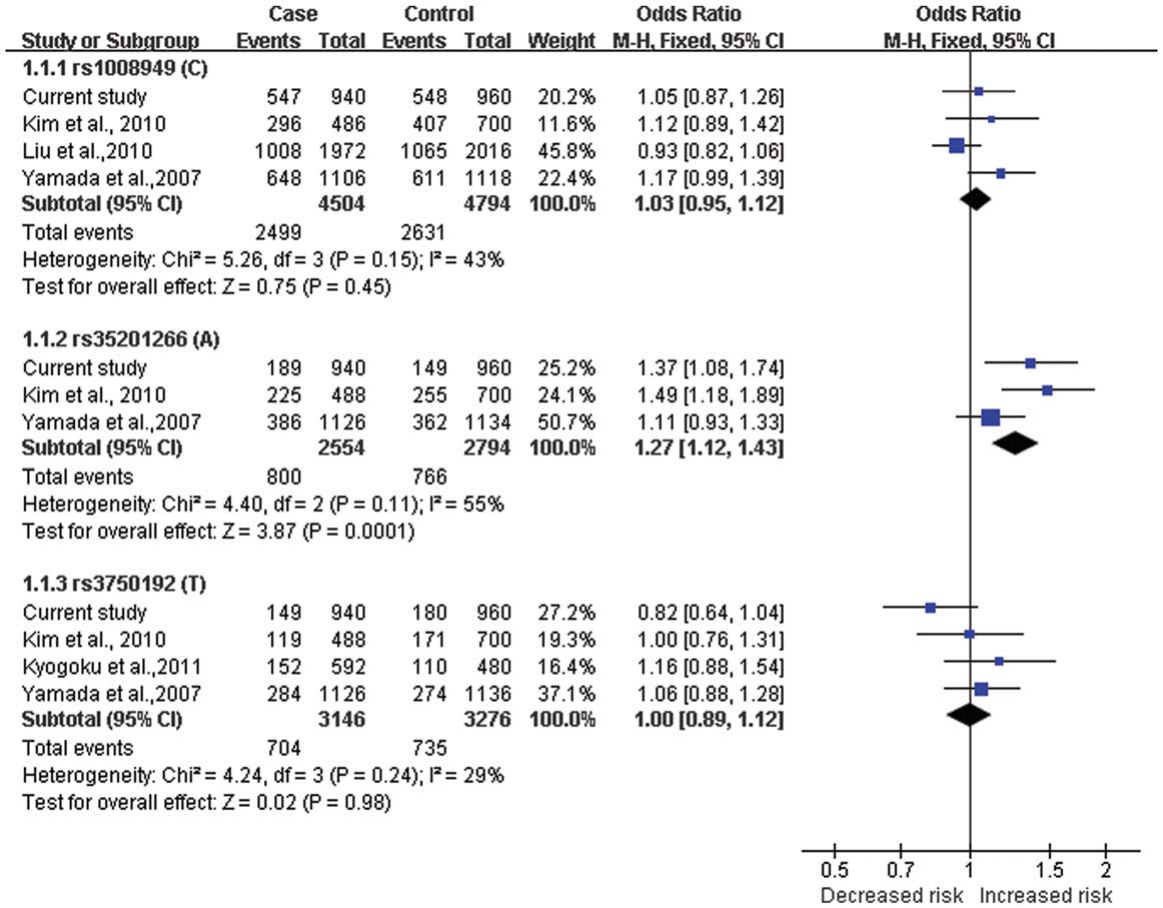
Meta-analysis of case-control studies between *EGR3* gene and schizophrenia.

For the meta-analysis, we combined and analyzed data from six populations (including this study). We utilized the schizophrenia research forum (http://www.szgene.org) and identified five independent case-control studies that tested for associations between various SNPs of *EGR3* and schizophrenia [8], [11], [18], [19], [26]. The study of Mansour *et al*. [26] did not provide enough information to calculate an effect of size, and therefore, was excluded from further analysis. We combined SNP data from the remaining studies and separately examined three SNPs (rs1008949, rs35201266 and rs3750192) in the *EGR3* gene locus. Summary statistics of the meta-analysis for these three SNPs are shown in Figure 3.

We tested for heterogeneity for each SNP of the combined sample, and the results were not significant [rs1008949 (*X* ^2^ = 5.26, *df* = 3, *P* = 0.15), rs35201266 (*X* ^2^ = 4.40, *df* = 2, *P* = 0.11) and rs3750192 (*X* ^2^ = 4.24, *df* = 3, *P* = 0.24)], enabling us to test for association of each of the SNPs by using a fixed-effect meta-analysis. A significant difference was found between patients and controls for the A-allele of rs35201266 (Subtotal OR = 1.27, 95%CI = 1.12–1.43, Z = 3.87, *P* = 0.0001), but not for rs1008949 (Subtotal OR = 1.03, 95%CI = 0.95–1.12, Z = 0.75, *P* = 0.45) and rs3750192 (Subtotal OR = 1.0, 95%CI = 0.89–1.12, Z = 0.02, *P* = 0.98).

Since only one family-based association study yielded a significant association (*P* = 0.0009) [8], we didn't perform a family-based meta-analysis.

## Discussion

In the current study, we found that SNP rs35201266 of *EGR3* and several haplotypes comprised of rs35201266 were significantly associated with schizophrenia patients of *Han* Chinese origin. Our SNP result is consistent with the findings of previous studies that were performed utilizing a Japanese sample of family-trios and a Korean case-control sample [8], [11]. A comparison of our results with the previous studies indicated that the A-allele of rs35201266 may be a risk allele for schizophrenia in Chinese, Korean, and a part of Japanese population. We also found that the MAF of rs35201266 was lower in the population we studied compared to other populations. This difference may be due to differences in ethnic backgrounds [27].

In the genetic model analysis, SNP rs35201266 showed strongly significant differences between cases and controls both in the additive model analysis (*P* = 0.0078) and the allelic comparisons (*P* = 0.009). Together with the weakly significant *P* value in the dominant model (*P* = 0.0258) and the recessive model (*P* = 0.0224), there is a reasonable interpretation for these findings: the additive model or the multiplicative model (the allelic comparisons) may be more accurate to describe the effect of *EGR3* in schizophrenia etiology [28]. Actually, the ORs for different models for the A-allele of rs35201266 are in the same direction, suggesting the increased risk of schizophrenia with the possession of one or more A alleles.

Furthermore, we stratified the patients by AAO, to examine if there were any clinical differences between the patients carrying the different allele of rs35201266. No significant differences were found between the risk allele (A) carriers and the G homozygotes of rs35201266 in either the total or the subgroup allele frequency distribution. Hence, our current study did not indicate an AAO specific genetic effect.

We also stratified samples by gender, however, there was no significant difference between males and females in cases (*P* = 0.0794). Thus, this result did not support a gender specific association for rs35201266 of *EGR3* with schizophrenia [29].

Our independent replication of the association of SNP rs35201266 with schizophrenia, in consideration with similar studies, reduces the likelihood that the association we observed was a false-positive and suggests that *EGR3* gene may indeed be a risk factor for schizophrenia. Recently, the genome-wide association studies (GWASs) are being widely used to study schizophrenia because they incorporate a powerful, systematic, and unbiased genetic approach to the analysis of complex diseases [2]. We summarized the findings of several GWASs that have detected associations with genetic variants in the vicinity of chromosome 8p, the same region that contains *EGR3*, and schizophrenia. Interestingly, the positive association between chromosome 8p and schizophrenia was identified at least in four different ethnic populations from three GWASs [23], [24], [25], however, the most significant candidate gene was not *EGR3*. Since the variants of *EGR3* locus may not surpass the level of genome-wide significance in GWA studies [30], the susceptibility of *EGR3* to schizophrenia cannot be excluded by GWASs. Hence, much work is still to be done to identify the contribution of *EGR3* to the involvement in genetic risk for schizophrenia.

To further confirm or exclude the implication of *EGR3* in association with schizophrenia, we performed a meta-analysis. We did not detect significant heterogeneity between the available studies, and this enabled us to test three SNPs (rs1008949, rs35201266 and rs3750192) for association with schizophrenia using a fixed-effects meta-analysis. We found a statistically significant association between rs35201266 and schizophrenia (*P* = 0.0001) in various East Asian populations. The estimates of the combined OR of rs35201266 ranged from 1.12 to 1.43, suggesting that the data from Kim's study [11] and our study affected the combined estimate, whereas, the study of Yamada *et al*. also showed an increase of risk allele in cases compared to controls (34.3% vs 31.9%) [8]. The results of our meta-analysis suggest that *EGR3* may have a small, but significant effect in the susceptibility to the development of schizophrenia, at least in the tested East Asian populations [31].

Specifically, we observed that the A-allele of rs35201266, and haplotypes comprised of the A-allele, were over-represented in patients with schizophrenia. As mentioned earlier, rs35201266 (A/G) is located in intron 1 of *EGR3*. In an in vitro study, the A-allele was associated with reduced expression of *EGR3*, suggesting that its function may be regulatory [8]. Reduced expression of *EGR3* has also been observed in patients with schizophrenia [8], [17]. Hence, the findings of our study are consistent with previous studies. Therefore, our study provides additional support for the possibility of SNP rs35201266 possessing a biological function in schizophrenia susceptibility.

*EGR3* may play a very important role in the pathological mechanism underlying schizophrenia. *EGR3*−/− mice were reported to display heightened reactivity to stress and novelty, abnormalities in social interactions, and deficits in synaptic plasticity, which model the cognitive deficits of schizophrenia [16]. The impact of the antipsychotic medication cloazpine inhibiting the increased aggression and impulsivity behavior of *EGR3*−/− mice is very similar with the effects of antipsychotic medications in schizophrenia patients [32].

In addition to EGR3's essential function for neuron activity, EGR3 also interacts with a number of factors implicated in the risk and pathogenesis of schizophrenia. Such factors include proteins and microRNAs (miRNAs) that have been reported to-affect/be-affected by the expression of *EGR3*. Proteins that have been reported to affect the expression of *EGR3* by induction include N-methyl-D-aspartate receptors (NMDARs), calcineurin (CN), brain-derived neurotropic factor (BDNF), and neuregulin (NRG1). EGR3 can be induced by NMDARs [33], which are highly permeable to Ca^2+^ (Ca^2+^ influx through NMDARs is essential for synaptic plasticity). Hypofunction of the NMDAR pathway has been reported to contribute to the etiology of schizophrenia in a number of studies [34], [35], [36].

EGR3 has been reported to be induced by CN [37], [38], [39]. CN is a Ca^2+^ and calmodulin-dependent phosphatase participating in many cellular processes and Ca^2+^-dependent signal transduction pathways [40]. Human genetic association studies and behavioral analysis of mouse models have provided evidence for the involvement of CN signaling in schizophrenia susceptibility [7], [41]. CN as an upstream gene for *EGR3* might be triggered by calcium influx through NMDARs [42]. *EGR3* is also a target gene for *Brain-derived neurotrophic factor* (BDNF) and *Neuregulin 1* (NRG1) [43], [44], [45], both of which are schizophrenia susceptibility genes [6], [46], [47], [48], [49]. Induction of *EGR3* by *BDNF* enables EGR3 to control the expression of *Gamma-aminobutyric acid receptor* (GABAR) [50], which is also a candidate risk gene for the schizophrenia [51].

In addition to being downstream and upstream of susceptibility genes, *EGR3* has been linked to a number of miRNAs that have been associated with schizophrenia. MiRNAs are a class of non-coding small RNAs that negatively regulate gene expression in numerous biological processes by promoting mRNA degradation and/or repressing translation through sequence-specific interactions with the 3′ UTRs of target mRNAs [52], [53]. The miR-15 family was reported to be significantly up-regulated in the cerebral cortex of schizophrenia patients and was therefore hypothesized to have a biological influence in the cortex of schizophrenia patients by influencing genes involved in cortical structure and neural plasticity [54]. *EGR3* is one of the targets genes for miR-15 family as predicated by the Miranda (http://www.microrna.org/microrna/home.do) and TargetScan (http://www.targetscan.org/) websites, consistent with the findings of elevated expression of miRNAs and decreased expression of *EGR3* in schizophrenia patients. Guo *et al.* proposed a model highlighting *EGR3* and miRNAs involved in signaling pathways and regulatory networks in the nervous system [55]. Further exploration regarding functional relationship between miR-15 family and *EGR3* gene involved in the pathogenesis of schizophrenia will be worthwhile.

We found that rs3750192 was not significantly associated with schizophrenia, in agreement with two other studies [11], [19] and in contrast to another study [8]. Yamada *et al.* utilizing a Japanese sample demonstrated that rs3750192 was associated with schizophrenia (*P* = 0.0171) [8]. The discrepancy of the results between studies may be due to different study designs and/or different ethnic groups. We also found that SNP rs1008949 was not associated with schizophrenia, consistent with the findings of another study (Liu et al.) utilizing a Chinese case-control sample [18].

The limitation of this study was that our sample was moderate in size and that there was not sufficient coverage of SNPs to span the whole sequence of *EGR3*. However, our results clearly demonstrate that SNP rs35201266 is significantly associated with schizophrenia, largely consistent with previous reports. We also showed that several haplotypes comprised of rs352021266 were significantly associated with schizophrenia.

In summary, our study provides evidence for the association of the *EGR3* locus and schizophrenia in the *Han* Chinese population. Considering the core position of *EGR3* in the CN signaling pathway, and its interaction with a number of candidate risk factors implicated with schizophrenia, it strongly warrants investigation of the molecular basis of *EGR3* in relationship to the pathogenesis of schizophrenia.

## Materials and Methods

### Subjects

The study was approved by the genetic research ethics committees of Xi'an Jiaotong University School of Medicine. The informed consent was written and obtained from all participants. Subjects of the case-control samples consisted of 470 patients with schizophrenia (245 males, mean age = 34.5±12.1, AAO = 24.2±7.2; 225 females, mean age = 32.0±13.9, AAO = 24.1±8.3) and 480 healthy control subjects (275 males, mean age = 29.4±14.3; 205 females, mean age = 29.6±14.1). All participants in this study were biologically unrelated individuals. The patients were diagnosed by the Psychiatry Department of the First Affiliated Hospital of Xi'an Jiaitong University School of Medicine according to the *Diagnostic and Statistical Manual of Mental Disorders* (DSM-IV) criteria for schizophrenia. The diagnosis was checked and confirmed by two independent senior psychiatrists who reviewed the psychiatric case records and excluded those with organic brain disease, or short-term drug-induced psychoses, or other symptomatic psychoses. The normal controls were drawn from a combination of local volunteers and blood transfusion donors. Subjects with a personal of family history of mental illness and with current or past evidence of psychoses were ruled out by psychiatric colleagues. All subjects were *Han* Chinese in origin.

### Genotyping

We selected four SNPs (rs1008949, rs35201266, rs3750192, and rs1877670) around the *EGR3* gene locus from the dbSNP database (http://www.ncbi.nlm.nih.gov/projects/SNP/) and the previous studies [8], [11]. These four SNPs span approximately 7.0 kb, with average interval of approximately 2.3 kb, and the density of SNPs is better than those GWASs using the 500 K chips (average interval ∼6.0 kb) [56]. In addition, two SNPs (rs35201266 and rs3750192) have been associated with schizophrenia in previous studies [8], [11]. The marker rs1008949 was studied both in Korean and Chinese populations [11], [18]. SNP rs1877670 was located in the 3′UTR of the *EGR3* gene and may play an important role in expression of *EGR3* [57]. DNA was extracted from whole blood according to a standard protocol of the DNA Isolation Kit for Mammalian Blood (Tiangen Biotech CO., LTD). Genotyping was accomplished by allele-specific PCR, methods have been described elsewhere [58]. PCR primers used in this study were designed by a tetra-primer ARMS-PCR primer design program (http://cedar.genetics.soton.ac.uk/public_html/primer1.html). The primers sequences are listed in Table S1. To ensure that the obtained genotypes were valid, re-genotyping was performed on 50 random DNA samples for each of the four SNPs. All genotypes were in agreement with the first round of genotyping, and no genotyping errors were found.

### Statistical Analysis

#### Primary analyses

Hardy-Weinberg equilibrium (HWE) and genetic models of all the SNPs were assessed using the software program Finetti (http://ihg2.helmholtz-muenchen.de/cgi-bin/hw/hwa1.pl). Genotype frequencies were analyzed using the software program Epi_Info (http://www.cdc.gov/epiinfo/). Allele frequencies and pair-wise marker LD were analyzed using the software program Haploview 4.1 [59]. Haplotype frequencies were estimated using the software program PHASE version 2.2 [60]. Rare haplotypes (i.e., found in less than 5% of both case and control subjects) were excluded from analysis. The distribution of global haplotype frequencies in cases and controls was compared using the software program Epi_Info. Bonferroni corrections were applied to all multiple statistical tests. The software program G*Power program [61] was used to determine statistical power of the case-control sample. Taking into account sample size, the case-control sample had >86% power to detect a significant association (a<0.05), when an effect size index corresponding to a “weak” effect (0.2) was used. Furthermore, we applied a more precise method (http://www.stat.ubc.ca/~rollin/stats/ssize/caco.html) to calculate our study power according to the ORs and MAFs of SNP rs35201266 reported by Kim *et al*., [11] and found that our current sample had >84% power to detect a significant association (a<0.05) (two sided test).

#### Secondary analyses

According to the genotyping data of rs35201266, the patients were separated into two groups: the risk allele (A) carriers [heterozygous and homozygous for the allele (A)] and non-risk allele carriers [homozygous for the allele (G)].To assess potential clinical differences (i.e., AAO and gender) in association, chi-squares, OR, and 95% CI for comparison between subgroups were calculated using the Epi_Info software. Furthermore, we compared our results with previous association studies.

#### Meta-analysis

The studies included in the meta-analysis were identified using Medline with the search terms ‘EGR3’ and ‘Schizophrenia’. All the data analyzed had been previously published. The significance of the subtotal OR was determined by Z test, and the heterogeneity of the group of ORs was assessed using a chi-square test. All statistical analyses were performed using the software program RewMan version 5.0 (http://www.cochrane.org/revman).

## Supporting Information

Table S1 **Markers and primers used for allele-specific PCR.**
(DOC)

## References

[1] Purcell SM, Wray NR, Stone JL, Visscher PM, O'Donovan MC (2009). Common polygenic variation contributes to risk of schizophrenia and bipolar disorder.. Nature.

[2] Riley B, Thiselton D, Maher BS, Bigdeli T, Wormley B (2009). Replication of association between schizophrenia and ZNF804A in the Irish Case-Control Study of Schizophrenia sample.. Mol Psychiatry.

[3] Badner JA, Gershon ES (2002). Meta-analysis of whole-genome linkage scans of bipolar disorder and schizophrenia.. Mol Psychiatry.

[4] Lewis CM, Levinson DF, Wise LH, DeLisi LE, Straub RE (2003). Genome scan meta-analysis of schizophrenia and bipolar disorder, part II: Schizophrenia.. Am J Hum Genet.

[5] Suarez BK, Duan J, Sanders AR, Hinrichs AL, Jin CH (2006). Genomewide linkage scan of 409 European-ancestry and African American families with schizophrenia: suggestive evidence of linkage at 8p23.3-p21.2 and 11p13.1-q14.1 in the combined sample.. Am J Hum Genet.

[6] Stefansson H, Sigurdsson E, Steinthorsdottir V, Bjornsdottir S, Sigmundsson T (2002). Neuregulin 1 and susceptibility to schizophrenia.. Am J Hum Genet.

[7] Gerber DJ, Hall D, Miyakawa T, Demars S, Gogos JA (2003). Evidence for association of schizophrenia with genetic variation in the 8p21.3 gene, PPP3CC, encoding the calcineurin gamma subunit.. Proc Natl Acad Sci U S A.

[8] Yamada K, Gerber DJ, Iwayama Y, Ohnishi T, Ohba H (2007). Genetic analysis of the calcineurin pathway identifies members of the EGR gene family, specifically EGR3, as potential susceptibility candidates in schizophrenia.. Proc Natl Acad Sci U S A.

[9] Lohoff FW, Weller AE, Bloch PJ, Buono RJ, Doyle GA (2008). Association between polymorphisms in the vesicular monoamine transporter 1 gene (VMAT1/SLC18A1) on chromosome 8p and schizophrenia.. Neuropsychobiology.

[10] Tabares-Seisdedos R, Rubenstein JL (2009). Chromosome 8p as a potential hub for developmental neuropsychiatric disorders: implications for schizophrenia, autism and cancer.. Mol Psychiatry.

[11] Kim SH, Song JY, Joo EJ, Lee KY, Ahn YM (2010). EGR3 as a potential susceptibility gene for schizophrenia in Korea.. Am J Med Genet B Neuropsychiatr Genet.

[12] Lee SM, Vasishtha M, Prywes R (2010). Activation and repression of cellular immediate early genes by serum response factor cofactors.. J Biol Chem.

[13] O'Donovan KJ, Tourtellotte WG, Millbrandt J, Baraban JM (1999). The EGR family of transcription-regulatory factors: progress at the interface of molecular and systems neuroscience.. Trends Neurosci.

[14] O'Donovan KJ, Levkovitz Y, Ahn D, Baraban JM (2000). Functional comparison of Egr3 transcription factor isoforms: identification of an activation domain in the N-terminal segment absent from Egr3beta, a major isoform expressed in brain.. J Neurochem.

[15] Li L, Yun SH, Keblesh J, Trommer BL, Xiong H (2007). Egr3, a synaptic activity regulated transcription factor that is essential for learning and memory.. Mol Cell Neurosci.

[16] Gallitano-Mendel A, Izumi Y, Tokuda K, Zorumski CF, Howell MP (2007). The immediate early gene early growth response gene 3 mediates adaptation to stress and novelty.. Neuroscience.

[17] Mexal S, Frank M, Berger R, Adams CE, Ross RG (2005). Differential modulation of gene expression in the NMDA postsynaptic density of schizophrenic and control smokers.. Brain Res Mol Brain Res.

[18] Liu BC, Zhang J, Wang L, Li XW, Wang Y (2010). No association between EGR gene family polymorphisms and schizophrenia in the Chinese population.. Prog Neuropsychopharmacol Biol Psychiatry.

[19] Kyogoku C, Yanagi M, Nishimura K, Sugiyama D, Morinobu A (2011). Association of calcineurin A gamma subunit (PPP3CC) and early growth response 3 (EGR3) gene polymorphisms with susceptibility to schizophrenia in a Japanese population.. Psychiatry Res.

[20] Lohmueller KE, Pearce CL, Pike M, Lander ES, Hirschhorn JN (2003). Meta-analysis of genetic association studies supports a contribution of common variants to susceptibility to common disease.. Nat Genet.

[21] Sham PC, MacLean CJ, Kendler KS (1994). A typological model of schizophrenia based on age at onset, sex and familial morbidity.. Acta Psychiatr Scand.

[22] Zhang F, Chen Q, Ye T, Lipska BK, Straub RE (2011). Evidence of sex-modulated association of ZNF804A with schizophrenia.. Biol Psychiatry.

[23] Shi J, Levinson DF, Duan J, Sanders AR, Zheng Y (2009). Common variants on chromosome 6p22.1 are associated with schizophrenia.. Nature.

[24] Yamada K, Iwayama Y, Hattori E, Iwamoto K, Toyota T (2011). Genome-wide association study of schizophrenia in Japanese population.. PLoS One.

[25] Ma X, Deng W, Liu X, Li M, Chen Z (2011). A genome-wide association study for quantitative traits in schizophrenia in China.. Genes Brain Behav.

[26] Mansour HA, Talkowski ME, Wood J, Chowdari KV, McClain L (2009). Association study of 21 circadian genes with bipolar I disorder, schizoaffective disorder, and schizophrenia.. Bipolar Disord.

[27] Bian L, Yang JD, Guo TW, Duan Y, Qin W (2005). Association study of the A2M and LRP1 Genes with Alzheimer disease in the Han Chinese.. Biol Psychiatry.

[28] Lewis CM (2002). Genetic association studies: design, analysis and interpretation.. Brief Bioinform.

[29] Zhang R, Zhong NN, Liu XG, Yan H, Qiu C (2010). Is the EFNB2 locus associated with schizophrenia? Single nucleotide polymorphisms and haplotypes analysis.. Psychiatry Res.

[30] Nieratschker V, Nothen MM, Rietschel M (2010). New Genetic Findings in Schizophrenia: Is there Still Room for the Dopamine Hypothesis of Schizophrenia?. Front Behav Neurosci.

[31] Ma J, Qin W, Wang XY, Guo TW, Bian L (2006). Further evidence for the association between G72/G30 genes and schizophrenia in two ethnically distinct populations.. Mol Psychiatry.

[32] Gallitano-Mendel A, Wozniak DF, Pehek EA, Milbrandt J (2008). Mice lacking the immediate early gene Egr3 respond to the anti-aggressive effects of clozapine yet are relatively resistant to its sedating effects.. Neuropsychopharmacology.

[33] Yamagata K, Kaufmann WE, Lanahan A, Papapavlou M, Barnes CA (1994). Egr3/Pilot, a zinc finger transcription factor, is rapidly regulated by activity in brain neurons and colocalizes with Egr1/zif268.. Learn Mem.

[34] Lau CG, Zukin RS (2007). NMDA receptor trafficking in synaptic plasticity and neuropsychiatric disorders.. Nat Rev Neurosci.

[35] Olney JW, Newcomer JW, Farber NB (1999). NMDA receptor hypofunction model of schizophrenia.. J Psychiatr Res.

[36] du Bois TM, Huang XF (2007). Early brain development disruption from NMDA receptor hypofunction: relevance to schizophrenia.. Brain Res Rev.

[37] Mittelstadt PR, Ashwell JD (1998). Cyclosporin A-sensitive transcription factor Egr-3 regulates Fas ligand expression.. Mol Cell Biol.

[38] Hildeman DA, Mitchell T, Kappler J, Marrack P (2003). T cell apoptosis and reactive oxygen species.. J Clin Invest.

[39] Droin NM, Pinkoski MJ, Dejardin E, Green DR (2003). Egr family members regulate nonlymphoid expression of Fas ligand, TRAIL, and tumor necrosis factor during immune responses.. Mol Cell Biol.

[40] Rusnak F, Mertz P (2000). Calcineurin: form and function.. Physiol Rev.

[41] Miyakawa T, Leiter LM, Gerber DJ, Gainetdinov RR, Sotnikova TD (2003). Conditional calcineurin knockout mice exhibit multiple abnormal behaviors related to schizophrenia.. Proc Natl Acad Sci U S A.

[42] Sheng M, Kim MJ (2002). Postsynaptic signaling and plasticity mechanisms.. Science.

[43] Roberts DS, Hu Y, Lund IV, Brooks-Kayal AR, Russek SJ (2006). Brain-derived neurotrophic factor (BDNF)-induced synthesis of early growth response factor 3 (Egr3) controls the levels of type A GABA receptor alpha 4 subunits in hippocampal neurons.. J Biol Chem.

[44] Hippenmeyer S, Shneider NA, Birchmeier C, Burden SJ, Jessell TM (2002). A role for neuregulin1 signaling in muscle spindle differentiation.. Neuron.

[45] Jacobson C, Duggan D, Fischbach G (2004). Neuregulin induces the expression of transcription factors and myosin heavy chains typical of muscle spindles in cultured human muscle.. Proc Natl Acad Sci U S A.

[46] Ashe PC, Berry MD, Boulton AA (2001). Schizophrenia, a neurodegenerative disorder with neurodevelopmental antecedents.. Prog Neuropsychopharmacol Biol Psychiatry.

[47] Wong J, Hyde TM, Cassano HL, Deep-Soboslay A, Kleinman JE (2009). Promoter specific alterations of brain-derived neurotrophic factor mRNA in schizophrenia.. Neuroscience.

[48] Alaerts M, Ceulemans S, Forero D, Moens LN, De Zutter S (2009). Support for NRG1 as a susceptibility factor for schizophrenia in a northern Swedish isolated population.. Arch Gen Psychiatry.

[49] Stefansson H, Sarginson J, Kong A, Yates P, Steinthorsdottir V (2003). Association of neuregulin 1 with schizophrenia confirmed in a Scottish population.. Am J Hum Genet.

[50] Roberts DS, Raol YH, Bandyopadhyay S, Lund IV, Budreck EC (2005). Egr3 stimulation of GABRA4 promoter activity as a mechanism for seizure-induced up-regulation of GABA(A) receptor alpha4 subunit expression.. Proc Natl Acad Sci U S A.

[51] Hashimoto T, Bazmi HH, Mirnics K, Wu Q, Sampson AR (2008). Conserved regional patterns of GABA-related transcript expression in the neocortex of subjects with schizophrenia.. Am J Psychiatry.

[52] Bartel DP (2004). MicroRNAs: genomics, biogenesis, mechanism, and function.. Cell.

[53] Bartel DP (2009). MicroRNAs: target recognition and regulatory functions.. Cell.

[54] Beveridge NJ, Gardiner E, Carroll AP, Tooney PA, Cairns MJ (2009). Schizophrenia is associated with an increase in cortical microRNA biogenesis.. Mol Psychiatry.

[55] Guo AY, Sun J, Jia P, Zhao Z (2010). A novel microRNA and transcription factor mediated regulatory network in schizophrenia.. BMC Syst Biol.

[56] O'Donovan MC, Craddock N, Norton N, Williams H, Peirce T (2008). Identification of loci associated with schizophrenia by genome-wide association and follow-up.. Nat Genet.

[57] Zhang R, Lu SM, Qiu C, Liu XG, Gao CG (2011). Population-based and family-based association studies of ZNF804A locus and schizophrenia.. Mol Psychiatry.

[58] Wang X, He G, Gu N, Yang J, Tang J (2004). Association of G72/G30 with schizophrenia in the Chinese population.. Biochem Biophys Res Commun.

[59] Barrett JC, Fry B, Maller J, Daly MJ (2005). Haploview: analysis and visualization of LD and haplotype maps.. Bioinformatics.

[60] Stephens M, Smith NJ, Donnelly P (2001). A new statistical method for haplotype reconstruction from population data.. Am J Hum Genet.

[61] Faul F, Erdfelder E, Lang AG, Buchner A (2007). G*Power 3: a flexible statistical power analysis program for the social, behavioral, and biomedical sciences.. Behav Res Methods.

