# Supplementary material for: Genetic Evidence for the Association between the Early Growth Response 3 (EGR3) Gene and Schizophrenia
Source: PLoS One. 2012 Jan 20;7(1):e30237. doi: 10.1371/journal.pone.0030237 (PMC3262808; doi:10.1371/journal.pone.0030237)
Supplement: Table S1 — Markers and primers used for allele-specific PCR. (DOC) [file pone.0030237.s001.doc]

**Table S1** Markers and primers used for allele-specific PCR

| **Marker** | **Physical** | **Primer Sequence** | | **Annealing** |
| --- | --- | --- | --- | --- |
|  | **Location** ^a^ | **Forward** ^b^ | **Reverse** | **temperature (℃)** |
| rs1008949 | 22,553,621 | F1: 5’- CAACTGGAGGAGACGGACT | R: 5’- CCCATGCTCTTCTACTTGGA | 56 |
|  |  | F2: 5’- CAACTGGAGGAGACGGACC |  |  |
| rs35201266 | 22,549,697 | F1: 5’- ACGGGGAACGATCCCGTGG | R: 5’- TGCTGGAGGGGAAAATCCTAGCC | 55 |
|  |  | F2: 5’- ACGGGGAACGATCCCGTGA |  |  |
| rs3750192 | 22,548,790 | F1: 5’- GTGGACGTCTGCGTGTTG | R: 5’- GACTCCCCTTCCAACTGGT | 55 |
|  |  | F2: 5’- GTGGACGTCTGCGTGTTT |  |  |
| rs1877670 | 22,546,561 | F1: 5’- TATATGTGTATATATGTACACTCAAAT | R: 5’- AGATTATATCTACATACGTGTGTTA | 56 |
|  |  | F2: 5’- TATATGTGTATATATGTACACTCAAAC |  |  |

^a^ UCSC Browser, Feb 2009; http://genome.ucsc.edu/cgi-bin/hgGateway.

^b^ An additional mismatch was deliberately put at position –3 from the 3’ terminus of the allele-specific primer to confer the specificity of PCR amplification.
